# Age Assessment in Children: A Novel Cameriere’s Stratagem

**DOI:** 10.5005/jp-journals-10005-1387

**Published:** 2016-12-05

**Authors:** Prabhakar Ramasetty Attiguppe, Chandrashekar Yavagal, Rekhamani Maganti, P Mythri

**Affiliations:** 1 Professor and Head, Department of Pedodontics and Preventive Dentistry, Bapuji Dental College and Hospital, Davangere, Karnataka, India; 2 Reader, Department of Pedodontics and Preventive Dentistry, Bapuji Dental College and Hospital, Davangere, Karnataka, India; 3 Postgraduate Student, Department of Pedodontics and Preventive Dentistry, Bapuji Dental College and Hospital, Davangere, Karnataka, India; 4 Senior Lecturer, Department of Pedodontics and Preventive Dentistry, Bapuji Dental College and Hospital, Davangere, Karnataka, India

**Keywords:** Davangere population, Open apices, Panoramic radiograph, Regression formula.

## Abstract

**Aim:**

Age is one of the essential factors in establishing the identity of a person, especially in children. Age estimation plays an important part in treatment planning, forensic dentistry, legal issues, and paleodemographic research. The present study was an attempt to estimate the chronological age in children of Davangere population by using Cameriere’s India specific formula.

**Materials and methods:**

This was a retrospective observational study to estimate the chronological age in children of Davangere population. A total of 150 panoramic radiographs of patients aged between 6 and 15 years, including both sexes, were selected. Age was calculated by measuring open apices of seven right or left mandibular teeth using Adobe Photoshop software.

**Results:**

Statistical analysis was performed to derive a regression equation for estimation of age, which showed that, of the variables *X_1_, X_2_, X_3_, X_4_, X_5_, X_6_, X_7_, s, N_0_,* the variables *N_0_* and X_4_ were statistically noteworthy. Hence, these two variables were used to derive the linear regression formula:

Age = 10.522 + 0.712(N_0_) - 5.040(X_4_). The model was found to be statistically significant, F(2, 147) = 207.96, p < 0.001, and accounted for approximately 74% of the variance of age (R^2^ = 0.739, adjusted R^2^ = 0.735).

**Conclusion:**

Cameriere’s method can be used for age assessment in children for forensic as well as legal contexts and based on these variables a reliable age estimation equation could be proposed specifically for Davangere population.

**How to cite this article:**

Attiguppe PR, Yavagal C, Maganti R, Mythri P. Age Assessment in Children: A Novel Cameriere’s Stratagem. Int J Clin Pediatr Dent 2016;9(4):330-334.

## INTRODUCTION

Forensic odontology is a vital and integral part of forensic science that is widely used for identification of the living and deceased persons.^1^ Age estimation plays an important role in pediatric endocrinology and developmental disorders. Clinical dentistry is for orthodontic diagnosis and treatment planning. Age estimation plays a vital role even outside medicine and dentistry. Some of its applications pertain to the current issues which are plaguing a country like India, viz., high rate of child marriages, increasing number of child laborers, and children trafficked for commercial and sexual exploitation. Most children are forced into these activities and to say that age estimation of such children is crucial would be an understatement.^2^

During the growth of a person the application of skeletal, odontological, anthropological, and psychological methods allows an appropriate assessment of age.^3^ However, the most frequently used skeletal methods present some drawbacks in view of the important variability of bone maturation which is influenced by environmental factors and high radiation dose. An alternative approach based on dental development is deemed suitable, especially in children, because calcification rate is controlled more by genes than environmental factors and therefore, yields low variability.^4-6^

A number of methods have been proposed to determine dental age, but the system developed by Demirjian has gained wide acceptance. Two studies testing the applicability of this method in Indian population showed an overestimation of age and concluded that it cannot be applied for Indian children.^7,8^ In 2006, Cameriere et al developed a new method for assessing chronological age in children based on the relationship between age and measurement of open apices in teeth.^3^ A study was conducted by using the aforementioned method and a Cameriere’s formula specific for Indian children was established.^9^

However, India being a large country with diverse lifeforms and a population of mixed ethnicity, it is imperative to derive region-specific formula. Hence the present study was designed to evaluate the viability of Cameriere’s specific formula for Davangere, India population.

## RESEARCH HYPOTHESIS

- There will be a correlation between the age estimated by measuring the open apices of teeth and the chronological age of the patient.

## MATERIALS AND METHODS

The study was conducted at the Department of Pedodon-tics and Preventive Dentistry, Bapuji Dental College and Hospital, Davangere in association with Department of Oral Medicine and Radiology, Bapuji Dental College and Hospital, Davangere. Approval from the Ethical Committee of the institution was obtained and the study was designed in accordance to the revised Helsinki Declaration (2013).

A total of 150 Orthopantomographs (OPGs) of age groups 6 and 15 years comprising both sexes were selected as per the inclusion criteria, for each interval of one year, i.e., 6 to 7, 8 to 9, 10 to 11, 12 to 13, 14 to 15. So, 30 OPGs were included.

## Study Design

It was a retrospective (observational study) study in which OPGs of individuals with the following criteria were included in the study:

- Patients of age group 6 to 15 years
- All teeth on right or left lower jaw should be present
- Should belong to Davangere district.

Individuals with the following conditions were excluded from the present study:

- Presence of third molars (as they were not studied)
- Radiographs showing any pathology on the concerned side of lower jaw
  Example: developmental abnormalities, grossly decayed teeth, tooth fracture, cysts, or tumors.
- Patients with previous history of orthodontic treatment.

The procedural steps were as follows:

For each individual the chronological age was calculated by subtracting the birth date from the date on which radiograph was exposed for that particular patient. The images were processed using a computer-aided drafting program, Adobe Photoshop. With the help of Adobe Photoshop, the following parameters were calculated with the measuring tool present on the drop-down column on the left-hand side of the screen.

**Fig. 1:**
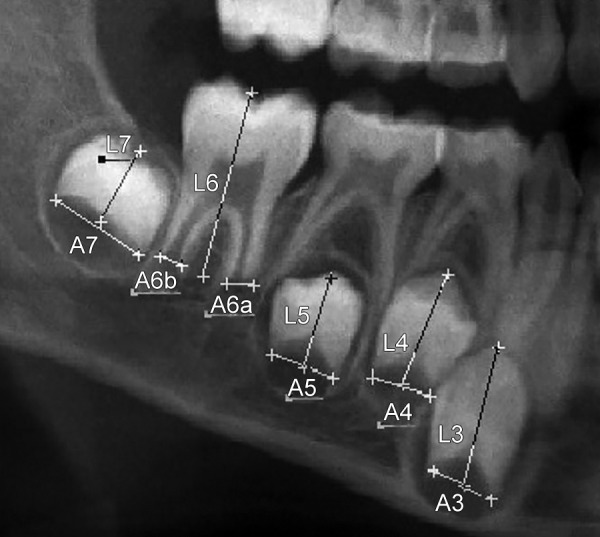
An example of tooth measurement in Adobe Photoshop. Ai, i = 1, . . . , 5 (teeth with one root), is distance between inner sides of open apex; Ai, i = 6 and 7 (teeth with two roots), is sum of distances between inner sides of two open apices; and Li, i = 1, . . ., 7, is length of seven teeth

- Length of the tooth and the distance between open apices
- The number of right or left mandibular teeth with root development complete, apical ends of roots completely closed were calculated and denoted as (N_0_). In teeth with incomplete root development and, therefore, with open apices, the distance between inner sides of open apex was measured. For teeth with one root the distance between inner sides of open apices was measured and denoted as *A_i_* where (i = 1, 2, 3, 4, 5): 1 - central incisor, 2 - lateral incisor, 3 - canine, 4 - first premolar, 5 - second premolar. For teeth with two roots, the sum of distance between inner sides of two open apices was calculated and denoted as *A_i _*(i = 6, 7); 6 - first molar, 7 - second molar (Fig. 1).

Due to possible difference in magnification and angulation among X-ray measurements, (A_i_) was normalized by dividing it with tooth length. Tooth length was measured from point of highest cusp to the root apex and was denoted as (L_i_) (Fig. 1).

Dental maturity was evaluated using normalized measurements of seven right or left mandibular teeth (X_i _= A_i_/L_i_); *i* = 1 to 7.

Therefore, sum of normalized open apices was calculated as *s* = X_1_ + X_2_ + X_3_ + ... + X_7_.

Age was estimated using the formula:

Age = 9.402 - 0.879C + 0.663N_0_ - 0.711s - 0.106s(N_0_)

where *C* is the variable, which is 0 for Central and North India and 1 for South India.

It was substituted as 1, since Davangere belongs to South India.

Measurement was carried out by single calibrated observer to rule out intraobserver variability. A random sample of OPGs was reexamined after an interval of at least 2 weeks and was subjected to statistical analysis.

## Statistical Analysis

For each sample, all the morphological variables, *X_i_, i* = 1-7, s, *N_0_,* and gender, were entered in an EXCEL sheet, so as to use them as predictive variables for age estimation in subsequent statistical analysis. Chronological age, calculated by subtracting the date of the radiograph from the date of birth, was also recorded. The intraobserver reliability of the sum of normalized open apices(s) was studied by means of the intraclass correlation coefficient. Kappa statistics were used to measure the intraobserver reliability of the number of the seven right or left permanent mandibular teeth with root development complete (N_0_). Furthermore, correlation coefficients were evaluated between age and predictive variables. A multiple linear regression model was developed by selecting those variables that contributed significantly to age estimation using the stepwise selection method.

**Table 1:** **Table 1:** Reliability analysis for various variables using Intra class correlation coefficient

		*Intraclass* *correlation*		*95% Confidence* *interval (CI)*	
X_1_		0.863		0.687-0.944	
X_2_		0.840		0.640-0.933	
X_3_		0.730		0.434-0.883	
X_4_		0.901		0.767-0.959	
X_5_		0.890		0.743-0.955	
X_6_		0.960		0.901-0.984	
X_7_		0.923		0.816-0.969	
s		0.992		0.980-0.997	

Kappa statistic for No is κ = 1

## RESULTS

There were no statistically significant intraobserver differences between the paired sets of measurements carried out on the reexamined panoramic radiographs. In fact, in the seven right or left permanent mandibular teeth with root development complete (N_0_), no misfit was observed between the two measurements made by the observer after 2 weeks, i.e., κ = 1 (Table 1).

When all the variables were entered in the EXCEL file for statistical analysis, it was found that of the variables X_i_, *i* = 1-7, *s, N_0_,* the variables *N_0_* and X_4_ were statistically noteworthy and contributed significantly to the fit (Table 2). These two variables were used to derive the linear regression formula:

Age = 10.522 + 0.712(N_0_) - 5.040(X_4_)        (1)

The model was statistically significant, F(2, 147) = 207.96, p < 0.001, and accounted for approximately 74% of the variance of age (R^2^ = 0.739, adjusted R^2^ = 0.735) (Table 3).

**Table 2:** **Table 2:** Correlation between dependent and independent variables in the study

		*Chronological age*		N0		X_1_		X_2_		X_3_		X_4_		X_5_		X_6_		X_7_		s	
Chronological		1.000																			
N_0_		0.801*		1.000																	
X_1_		–0.698*		–0.676*		1.000															
X_2_		–0.642*		–0.640*		0.867*		1.000													
X_3_		–0.646*		–0.656*		0.579*		0.585*		1.000											
X_4_		–0.794*		–0.721*		0.737*		0.763*		0.832*		1.000									
X_5_		–0.715*		–0.683*		0.673*		0.758*		0.706*		0.863*		1.000							
X_6_		–0.620*		–0.570*		0.823*		0.833*		0.594*		0.761*		0.697*		1.000					
X_7_		–0.666*		–0.646*		0.619*		0.586*		0.583*		0.735*		0.688*		0.618*		1.000			
*s*		–0.810*		–0.766*		0.811*		0.828*		0.798*		0.942*		0.885*		0.825*		0.811*		1.000	
SEX		0.121		0.096		–0.117		–0.132		–0.080		–0.150*		–0.132		–0.158*		–0.048		–0.139*	

**Table 3:** **Table 3:** Regression analysis

										*Change statistics*									
*Model*		*R*		R^2^		*Adjusted R^2^*		*Std. error of* * the estimate*		*R^2^ change*		*Fchange*		*dfl*		*df2*		*Sig. F change*	
1		0.810^a^		0.656		0.654		1.6888940		0.656		282.500		1		148		0.000	
2		0.857^b^		0.735		0.731		1.4883219		0.079		43.578		1		147		0.000	
3		0.862^c^		0.743		0.738		1.4690776		0.009		4.877		1		146		0.029	
4		0.860^d^		0.739		0.735		1.4769329		-0.005		2.576		1		146		0.111	
	
				Unstandardized coefficients							Standardized coefficients								
*Model*				*B*					*Std. error*			*Beta*			*t*				*p-value*
(Constant)				10.522					0.376						27.948				<0.01*
^N^0				0.712					0.091			0.476			7.833				<0.01*
^X^4				-5.040					0.680			-0.450			-7.409				<0.01*

R^2^ = 0.739, Adjusted R^2^ = 0.735, F(2, 147) = 207.96, *p < 0.01

## DISCUSSION

Accurate age estimation is an important issue for human identification, both for human remains as well as for living individuals.^10^ Age estimation is important from a forensic perspective as well and is especially useful in establishing the difference between the juvenile and the adult states of an individual in law suites. Assigning an age to a living child of unknown identity may be absolutely necessary when the child is the victim of a crime, suspected of a crime (when penal codes differentiate law and punishment for children of different ages), or when the child is a refugee of uncertain age, as well as in situations where the individuals do not have valid documentation of age. In these cases, it is imperative to employ noninvasive methods with higher accuracy and precision, because of specific legal requirements.^11^

Chronologic age estimation by tooth development has been used since a long period. Amongst several recognized factors that play a role in human growth, maturation, and body composition, ethnicity or race is considered to be a major influence altering growth and accounting for differences between groups.^12^ The study of the morphological parameters of teeth on dental X-rays of adult humans is more reliable than most other methods of age estimation and is thus commonly employed to determine age in living humans.^3^ Several studies show that morphological measurements can be reliably obtained on panoramic radiographs, provided some corrections are made to take into account the individual variability of tooth size as well as differences in magnification of radiographs and angulation between the X-ray beam and film.^3^ However, the various methods of age estimation based on the teeth do not provide a common formula for the whole world. To ameliorate the accuracy of dental age estimation procedures, it is necessary to collect referral databases of panoramic radiographs of the same national and ethnic origin and derive region-specific formulae for accurate age estimation. ^13^

In the present study, the India-specific formula could not be applied to Davangere samples as India happens to be a large country with diverse lifeforms and a population of mixed ethnicity. Thus for the present intervention, it was made sure that the samples included OPGs of individuals from Davangere district with at least the last two generations residing in Davangere itself. This was done to ensure ethnic uniformity of the study samples, considering the fact that the development of teeth varies among populations and is genetically determined.

In the present study, estimation of chronological age was performed by measuring the open apices of the teeth excluding third molars of healthy children aged between 6 and 15 years. The panoramic radiograph was specifically chosen as it is considered the best tool for age estimation in children because intraoral radiography is difficult to obtain in them without image distortion.^13^ Moreover, panoramic radiographs are ideal screening tools as they are inexpensive, readily available, provide an unobstructed view of the entire dental arch and have less radiation exposure. The maxillary teeth were not included as their apices are often obscured by super-imposition from anatomical landmarks or errors from the radiographic procedures.

When open apices were measured on digital panoramic images of mandibular teeth, it was shown that there were no significant intraobserver differences. Based on this, we could conclude that although the present technique involved more steps during calculation, it was faster and easier than other quantitative methods, thus paving a way for reliable and reproducible measurements.

Our study showed no statistical difference in dental age between male and female samples, which proves the fact that gender does not influence age estimation, thus validating the former’s exclusion in the original model equation as well. However, it is very likely that the maturation of girls and boys in India may occur at about the same time because the early maturation of girls may be offset by malnutrition and the greater amount of physical work required of them.^13^

In the present study the prediction model contained two of the ten predictors and was reached in four steps with one variable excluded. The model was statistically significant, F(2, 147) = 207.96, p < 0.001, and accounted for approximately 74% of the variance of age (R^2^ = 0.739, adjusted R^2^ = 0.735). Age was primarily predicted by lower values of first premolar and to a lesser extent by higher values of *N_0_.* This is in accordance to previous studies and hence these parameters were included in the regression equa-tion.^9^ Our method, based on the normalized open apices of the seven left or right permanent mandibular teeth, employed a second-degree polynomial function [Eq. (1)]. The results found in this work, like those of others,^14^ indicate that more attention should be focused on the possible differences between children of different origins.

The limitations of the study are that the derived regression equation does not differentiate between fast-or slow-maturing children.

## CONCLUSION

The present research has confirmed that there is significant correlation between age and measurement of open apices of teeth. This method can be used for assessing age in forensic as well as legal contexts, and based on these variables, a reliable age estimation equation can be proposed for the Davangere population.
